# Prevalence and Risk Factors for Vitamin C Deficiency in North and South India: A Two Centre Population Based Study in People Aged 60 Years and Over

**DOI:** 10.1371/journal.pone.0028588

**Published:** 2011-12-06

**Authors:** Ravilla D. Ravindran, Praveen Vashist, Sanjeev K. Gupta, Ian S. Young, Giovanni Maraini, Monica Camparini, R. Jayanthi, Neena John, Kathryn E. Fitzpatrick, Usha Chakravarthy, Thulasiraj D. Ravilla, Astrid E. Fletcher

**Affiliations:** 1 Aravind Eye Hospital Pondicherry, Pondicherry, India; 2 Dr. Rajendra Prasad Center for Ophthalmic Sciences, All India Institute of Medical Sciences, New Delhi, India; 3 Centre for Public Health, School of Medicine, Dentistry and Biomedical Sciences, Queen's University Belfast, Belfast, United Kingdom; 4 Dipartimento di Scienze Otorino-Odonto-Oftalmologiche e Cervico Facciali, Sezione di Oftalmologia, Università degli Studi di Parma, Parma, Italy; 5 Faculty of Epidemiology and Population Health, London School of Hygiene and Tropical Medicine, London, United Kingdom; 6 Centre for Vision and Vascular Science, School of Medicine, Dentistry and Biomedical Sciences, Queen's University Belfast, Belfast, United Kingdom; 7 Lions Aravind Institute of Community Ophthalmology, Madurai, India; Kenya Medical Research Institute - Wellcome Trust Research Programme, Kenya

## Abstract

**Background:**

Studies from the UK and North America have reported vitamin C deficiency in around 1 in 5 men and 1 in 9 women in low income groups. There are few data on vitamin C deficiency in resource poor countries.

**Objectives:**

To investigate the prevalence of vitamin C deficiency in India.

**Design:**

We carried out a population-based cross-sectional survey in two areas of north and south India. Randomly sampled clusters were enumerated to identify people aged 60 and over. Participants (75% response rate) were interviewed for tobacco, alcohol, cooking fuel use, 24 hour diet recall and underwent anthropometry and blood collection. Vitamin C was measured using an enzyme-based assay in plasma stabilized with metaphosphoric acid. We categorised vitamin C status as deficient (<11 µmol/L), sub-optimal (11–28 µmol/L) and adequate (>28 µmol/L). We investigated factors associated with vitamin C deficiency using multivariable Poisson regression.

**Results:**

The age, sex and season standardized prevalence of vitamin C deficiency was 73.9% (95% confidence Interval, CI 70.4,77.5) in 2668 people in north India and 45.7% (95% CI 42.5,48.9) in 2970 from south India. Only 10.8% in the north and 25.9% in the south met the criteria for adequate levels. Vitamin C deficiency varied by season, and was more prevalent in men, with increasing age, users of tobacco and biomass fuels, in those with anthropometric indicators of poor nutrition and with lower intakes of dietary vitamin C.

**Conclusions:**

In poor communities, such as in our study, consideration needs to be given to measures to improve the consumption of vitamin C rich foods and to discourage the use of tobacco.

## Introduction

Vitamin C (ascorbic acid) plays a major role in human metabolism ranging from the synthesis of collagen, carnitine and norepinephrine to a large number of antioxidant activities [1]. Humans are unable to synthesize vitamin C and are dependent on dietary sources, mainly fruit and vegetables. Even in high income countries population-based studies have reported blood levels of vitamin C in the range indicating deficiency in around 1 in 5 men and 1 in 9 women in low income groups [2], [3], [4], [5], [6]. Smokers, non-users of vitamin supplements and those consuming less than the recommended dietary intakes of vitamin C were subgroups shown to be at higher risk in these studies. In the US where supplement use is common among older people, vitamin C deficiency is lower than in younger age groups [3], [5], but in the UK with a lower use of supplements, higher vitamin C deficiency and lower dietary intakes [7] have been reported in older people. There are scarce data on vitamin C deficiency in resource-poor countries where vitamin C deficiency might be expected to be more prevalent. A systematic review found that a third of reproductive age women in resource-poor settings had dietary vitamin C intakes below the Estimated Average Requirements (EAR) rising to nearly 50% in Africa and South East Asia [8]. The limited data on older people in India show high rates of malnutrition [9]. Tobacco use is common in India with a third of adults smoking or chewing tobacco [10] . These factors: poor diets, especially in the older age group, and high use of tobacco suggest that vitamin C deficiency might be high in the older Indian population but currently no studies have investigated this or whether risk factors for vitamin C deficiency differ from those reported in high income populations. We aimed to estimate the prevalence of vitamin C deficiency in an older population in India and examine the factors associated with deficiency.

## Methods

### Ethics Statement

Participants gave full informed written consent. Illiterate subjects had the information leaflet read out to them and provided a thumb impression. The study complied with the guidelines in the Declaration of Helsinki and ethics approval was received from the Indian Council for Medical Research, Research Ethics Committees of the All India Institute of Medical Sciences, Aravind Eye Hospital, London School of Hygiene and Tropical Medicine and Queen's University Belfast.

### Study Population

The India age-related eye disease study (INDEYE) is a population-based study in two geographically different locations in India. The study sampling has been described in detail elsewhere [11]. We aimed to enroll 3000 people aged ≥60 years in each of the two study locations allowing for a response rate of 80%. The sample size calculations were based on the estimated prevalence of age-related macular degeneration. People aged 60 and over were identified from household enumeration of randomly sampled clusters in Gurgaon district, Haryana state, north India, and Pondicherry and Cuddalore district in Tamil Nadu, south India. These areas were chosen to represent a mix of rural and urban populations served by the participating eye hospitals (Dr Rajendra Prasad Center of Ophthalmic Sciences, (RPC), All India Institute of Medical Sciences, Delhi and the Aravind Eye Hospital (AEH), Pondicherry). Gurgaon city and Pondicherry city were excluded due to the high mobility in and out of these two locations. All enumerated people aged 60 and over were invited to take part in the study.

Data collection took place between September 2004 and December 2006. Enumerators collected household and individual socio-demographic and economic data. Fieldworkers interviewed participants at home with a structured questionnaire which included current and past tobacco use (smoking beedie (small hand rolled cigarettes) and/or cigarettes, chewing or inhaling), current and past alcohol use and type of cooking fuels. Diet was assessed by 24 hour recall. Within a week of the home interview participants were brought to the base hospital for the clinical examination which included anthropometry, an eye examination and blood sample collection. All examinations took place in the morning. Anthropometry included measurement of height, weight, and mid-upper arm circumference (MUAC). Participants were asked to remove heavy outer garments and take off their shoes. Standing height was measured to the nearest 0.1 cm using a portable stadiometer. Weight was measured using electronic scales and recorded to the nearest 0.1 kilogram. MUAC was measured at the mid-point between the inferior border of the acromion process (shoulder bone) and the tip of the olecranon process (elbow) to the nearest 0.1 cm on the bare left arm, using a fibre glass insertion tape. People were asked to bring medications or nutritional supplements to the hospital and details were recorded.

### Blood sample

For each participant a sample of 15 ml blood was collected in a shaded room in two different vacutainer tubes (10 ml clotted and 5 ml EDTA unclotted). The EDTA unclotted sample was kept in the refrigerator and processed within 2 hours after collection. The EDTA samples were centrifuged at 3000 rpm at 4°C (using a cold centrifuge) for 15 minutes. After centrifugation, exactly 100 µl of plasma were transferred to each of two storage tubes using a Merck graduated pipette and exactly 900 µl of 5% metaphosphoric acid (MPA) were added to each tube and the contents were mixed by gentle inversion without shaking. The aliquoted samples were kept in the fridge until the samples of the last participant of the morning had been processed and all samples were placed in the dedicated study freezer (−70°C) for storage until shipment. Fresh MPA solution was made up every two weeks by dissolving 5 g of metaphosphoric acid crystals in 100 ml of distilled water. The solution was placed in a dark glass bottle and kept in the refrigerator at 4°C. The median time of storage of blood samples was 1.1 years. Samples were subsequently shipped by air to Queen's University Belfast in dry ice using a courier service with tracking and monitoring of sample temperature throughout the shipping process. Vitamin C was measured by automated fluorimetric assay [12] on a Cobas FARA centrifugal analyzer (Roche Diagnostics, Switzerland). The limit of detection of the assay was 2 µmol/L.

Assays were standardised against the US National Institute of Standards and Technology (NIST) standard reference materials. We also collected from each participants a non-fasting sample of capillary blood which was assessed for glucose (CBG) using a reagent strip test and reflectance meter.

### Data preparation and Statistical Analysis

Nutrient intakes of energy and vitamin C were calculated from the individual food items in the 24 hour recall using the Indian food composition tables [13]. Dietary vitamin C was adjusted for total energy intake using the residual model of Willett [14]. Plasma vitamin C status was categorised as deficient (<11 µmol/L), sub-optimal (11–28 µmol/L) and adequate (>28 µmol/L). This categorisation was used by previous authors [2], [4] and based on expert committee recommendation [15]. Principal component analysis was used to derive a socio-economic status index (SES) (based on caste, landholding, type of roof and number of rooms in house). Current use of household cooking fuels was classified as clean (kerosene, electricity, LPG, Bio gas/gobar gas) or biomass (wood, crop residues, dung cakes). Alcohol and tobacco use were categorized as current, or never and past. Body Mass Index (BMI) was calculated as weight (kg)/ height (cm)^2^. Nutritional status was indicated by BMI and MUAC. We used WHO guidelines for categories of BMI in Asians with underweight defined as <18.5, normal weight as ≥18.5 to <25, and overweight and obese defined as ≥25 [16].

We defined MUAC values as normal (>23 in men and >22 in women) or mild malnutrition (22.1-23 in men and 20.1–22 in women) and moderate to severe malnutrition (<22 in men and <20 in women) [17]. Diabetes was defined as CBG ≥110 mg/dl.[18] We categorised season according to the India Meteorological Department classification, http://www.imd.gov.in/doc/climate_profile.pdf (accessed May 3^rd^ 2011) using the date of the clinical examination when the blood sample was collected.

Statistical analysis was carried out using Stata 11 (StataCorp. 2009. *Stata Statistical Software: Release 11*. College Station, TX: StataCorp LP.). We estimated the age, sex and season standardized prevalence of vitamin C status in the two study locations using the total study population as the standard. We undertook univariable and multivariable Poisson regression to investigate the association of factors expected *a priori* to be associated with plasma vitamin C status: age, sex, socio-economic status, biomass cooking fuels, tobacco and alcohol use, diabetes, anthropometry (BMI, MUAC), season and dietary vitamin C. In these models dietary vitamin C across the distribution of both locations was categorised by quartiles. We checked for interactions (p <0.05) by location for all variables in the model. Due to correlation between BMI and MUAC we included these variables separately in multivariable analysis. In all analyses we took account of the study design of cluster sampling using either the survey suite of commands (svy) in Stata to compute standard errors using linearized variance estimators or by the robust cluster option in Poisson regression.

## Results

Of 7518 enumerated people aged 60 years and over, 5900 (78%) attended the hospital-based clinical examination of whom 5702 (76%) gave a blood sample. Plasma vitamin C was available in 5638 (2668 from RPC and 2970 from AEH). Those without vitamin C data (non responders to the clinical exam and those with no blood sample) were older, 69.7 years (SD = 8) compared to those with vitamin C data, 67.6 years (SD = 6), p <0.00001. There were no differences by sex, socio-economic status (or its individual components of education, caste, or landholding) or by the season of enumeration of the villages. Dietary measures of vitamin C were available in 5502 of those with plasma vitamin C. Very few people (n = 69, 1.2%) reported taking any nutritional supplements and all of these were from south India. Thirty percent of the study population (n = 1692) had plasma levels below 2 µmol/L. The majority of people with levels below 2 (n = 1184) were from north India.

The age, sex and season standardized prevalence of vitamin C deficiency was 73.9% (95% Confidence Interval, CI 70.4, 77.5) in north India and 45.7% (95% CI 42.5, 48.9) in south India (Table 1). Only 10% of those in the north and a quarter of those in the south met the criteria for adequate levels. In both locations the prevalence of vitamin C deficiency was higher in men compared to women, and increased with increasing age (Table 2). There were significant associations across the three categories of vitamin C status (adequate, sub-optimal, deficient) with increasing age, lower proportions of women, higher proportions of the lowest socio-economic group, users of biomass cooking fuels, and current tobacco users (Table 3). The proportions of those with indices of poor nutrition (BMI of <18.5 or MUAC <22 in men and <20 in women) increased from adequate status to deficiency. Alcohol use was rarely reported by women (12 current and 6 past, 1.1% in the south and one past user in the north). There was no association between current alcohol consumption and vitamin C status in men. Being overweight (BMI > 25) was associated with better vitamin C status (Table 3). There was no association with diabetes. Dietary vitamin C intakes decreased with poorer plasma vitamin C status. This difference was most marked in the north, from 31.9 mg/day for those with adequate plasma vitamin C status to 19.5 for those categorized as deficient.

**Table 1:** Age, sex and season-standardized prevalence of plasma vitamin C adequacy status in people aged 60 years and over by location in India.

Plasma vitamin C	Adequate	Sub-optimal	Deficient
	>28 µmol/L	11–28 µmol/L	<11 µmol/L
North India			
N = 2668	n = 286	n = 403	n = 1979
Prevalence	10.8	15.3	73.9
95%CI	8.0, 13.5	13.8, 16.8	70.4, 77.5
South India			
N = 2970	n = 774	n = 853	n = 1343
Prevalence	25.9	28.4	45.7
95%CI	22.9, 28.9	26.3, 30.6	42.5, 48.9

**Table 2:** Age and sex specific prevalence of plasma vitamin C deficiency (<11 µmol/L) in people aged 60 years and over by location in India.

	North India		South India	
	N	Prevalence	N	Prevalence
		95% CI		95% CI
Men	1283	77.7	1407	51.4
		70.9, 84.5		47.4, 55.4
Women	1385	70.9	1563	39.7
		61.9, 79.8		33.9, 45.4
Age group				
60–64	985	68.7	1080	36.6
		60.1, 77.4		29.5, 43.7
65–69	658	72.2	864	39.3
		62.9, 81.6		32.2, 6.3
70–74	552	80.8	575	42.1
		73.7, 88.0		33.3, 50.9
75–79	287	79.0	275	44.6
		70.8, 87.2		34.2, 55.0
80+	186	85.0	176	50.7
		78.3, 91.7		38.2, 63.2

**Table 3:** Characteristics by plasma vitamin C status by location in India.

	North India				South India			
Plasma vitamin C	Adequate	Sub-optimal	Deficient	p	Adequate	Sub-optimal	Deficient	p
	>28 µmol/L	11–28 µmol/L	<11 µmol/L		>28 µmol/L	11–28 µmol/L	<11 µmol/L	
	N = 286	N = 403	N = 1979		N = 774	N = 853	N = 1343	
Age^1^	65.8 (5.9)	66.8 (6.1)	68.3 (6.8)	<0.0001	66.9 (6.2)	67.1 (6.1)	67.9 (6.6)	0.002
Women^2^	182 (63.6)	221 (54.8)	982 (49.6)	0.03	449 (58.0)	494 (57.9)	620 (46.2)	0.0001
Lowest SES^2,3^	41 (14.3)	77 (19.1)	463 (23.4)	0.02	139 (18.0)	181 (21.2)	346 (25.8)	0.02
Biomass fuels^2^	194 (67.8)	290 (72.0)	1641 (82.9)	0.001	349 (45.3)	448 (53.0)	706 (53.4)	0.1
Malnutrition^2,4^	22 (7.7)	46 (11.4)	343 (17.3)	0.01	72 (9.3)	104 (12.2)	254 (18.9)	<0.0001
Body mass Index								
<18.5 2	50 (17.5)	99 (24.6)	698 (35.5)	<0.0001	191 (24.7)	247 (29.1)	476 (35.8)	0.002
≥25^2^	55 (19.2)	74 (18.4)	233 (11.8)	0.003	178 (23.0)	171 (20.2)	206 (15.5)	0.003
Diabetes^2^	151 (52.8)	249 (61.8)	1141 (57.7)	0.1	447 (57.8)	459 (53.8)	755 (56.2)	0.3
Current tobacco 2	126 (44.1)	230 (57.1)	1284 (64.9)	<0.0001	248 (32.0)	363 (42.6)	709 (52.8)	<0.0001
Current alcohol 2,5	36 (34.6)	71 (39.0)	447 (44.8)	0.1	113 (34.8)	122 (34.0)	271 (37.5)	0.6
Dietary vitamin C^6^	31.9	29.9	19.5	<0.0001	35.6	35.0	33.2	0.02
	18.0, 50.9	17.5, 44.6	11.8, 33.5		24.7, 53.7	25.1, 49.4	22.9, 48.7	

1 Mean (Standard Deviation).

2 n with characteristic (%).

3 Socio-Economic status.

4 Moderate & severe malnutrition defined as a mid-upper arm circumference of <22 in men and <20 in women.

5 1283 men in north India and 1407 men in south India.

6 Median, (InterQuartile range) mg/day.

In Poisson regression comparing those with vitamin C deficiency with those with adequate levels, there were significant interactions with location and current tobacco use (p = 0.001) and location and season, p <0.0001) in univariable and multivariable analyses (Table 4). The association with tobacco use was stronger in the south (multivariable adjusted prevalence rate ratio, PRR 1.29, 95% CI 1.18, 1.41) compared to the north (PRR of 1.07, 95% CI 1.01, 1.13). The pattern of associations with season varied according to location. In north India, compared to the winter season (December to February) the months of June to September, and October to November were associated with a higher prevalence of deficiency (multivariable adjusted PRR of 1.27, 95% CI 1.16, 1.38 and 1.27, 95% CI 1.17, 1.38) respectively. In the south of India the months of March to May and June to September were associated with a lower prevalence of vitamin C deficiency, PRRs of 0.83 (95% CI 0.74, 0.94) and 0.73 (95% CI 0.61, 0.89) compared to the winter months. For other variables in the multivariable analysis significant associations with vitamin C deficiency remained for age, sex, use of biomass fuels, low BMI and dietary vitamin C with attenuation of the PRRS compared to the univariable analysis. Results were similar when MUAC was included in place of BMI (data not shown).

**Table 4:** Prevalence Rate Ratios for plasma vitamin C deficiency (<11 µmol/L) compared to adequate (>28 µmol/L).

	Univariable analysis			Multivariable analysis		
	PRR^1^	95% CI	p	PRR^2^	95% CI	p
Age Group						
60–64	1			1		
65–69	1.05	0.99, 1.10		1.07	1.02, 1.12	
70–74	1.11	1.05, 1.17		1.08	1.03, 1.14	
75–79	1.11	1.04, 1.19		1.09	1.02, 1.16	
80+	1.17	1.09, 1.25		1.14	1.07, 1.22	
P trend			<0.0001			<0.0001
Women	0.91	0.86, 0.96	<0.0001	0.93	0.89, 0.98	0.003
Lowest SES^3^	1.11	1.04,1 .18	<0.0001	1.03	0.99, 1.07	0.2
Biomass fuels	1.25	1.14, 1.20	<0.0001	1.03	0.98, 1.09	0.02
Malnutrition	1.13	1.07, 1.20	<0.0001			
Body mass Index						
<18.5	1.11	1.06,1 .16	<0.0001	1.05	1.03, 1.09	<0.001
≥18.5–<25	1			1		
≥25	0.91	0.84, 0.96	0.002	0.97	0.93, 1.02	0.3
Diabetes	1.02	0.97,1.07	0.6	1.01	0.97, 1.05	0.6
Current tobacco 4						
North India	1.09	1.02, 1.15	0.01	1.07	1.01, 1.13	0.02
South India	1.34	1.19, 1.50	<0.0001	1.29	1.18, 1.41	<0.0001
Dietary vitamin C^5^						
<18	1			1		
>18–29	0.88	0.83, 0.93		0.99	0.95, 1.03	
>29–44	0.80	0.76, 0.86		0.95	0.91, 0.99	
>44	0.73	0.68, 0.77		0.90	0.86, 0.94	
P trend			<0.0001			<0.0001
Season 6						
North India						
December to February	1			1		
March to May	1.11	0.96, 1.29	0.2	1.09	0.95, 1.25	0.2
June to September	1.33	1.21, 1.46	<0.0001	1.27	1.16, 1.38	<0.0001
October to November	1.27	1.16, 1.40	<0.0001	1.27	1.17, 1.38	<0.0001
South India						
December to February	1					
March to May	0.86	0.61, 0.91	0.03	0.83	0.74,0.94	0.003
June to September	0.74	0.61, 0.91	0.004	0.73	0.61,0.89	0.001
October to November	0.87	0.77, 0.99	0.04	0.91	0.78,1.06	0.2

1 Prevalence rate ratios adjusted for age and sex.

2 Prevalence rate ratios adjusted for variables in the Table.

3 Socio-Economic status.

4 interaction for tobacco use and vitamin C deficiency by location, p = 0.001.

5 Quartiles of dietary vitamin C (mg/day).

6 interaction for season and vitamin C deficiency by location, p <0.0001.

In multivariable Poisson regression of factors associated with sub-optimal compared to adequate vitamin C status, only tobacco use and, in the north, season were associated. The PRRs were similar to those reported for vitamin C deficiency (tables available from authors on request). There was no significant interaction between location and tobacco use. The smaller number in these analyses limited the power to investigate associations and interactions.

## Discussion

We found a high prevalence of vitamin C deficiency in older people in India; 74% of those in the north of India and 46% in the south of India were deficient and a further 15% and 28% respectively had sub-optimal levels. In common with other studies [3], [4], [5], [6] we found that vitamin C deficiency was more common in men and in users of tobacco. The lower levels of vitamin C in smokers partly reflect lower intakes but also a higher rate of ascorbate turnover possibly due to higher levels of oxidative stress in smokers [19]. The association with tobacco was observed in both south and north India but the PRR was higher in the south. There were differences in the pattern of tobacco use by location. Tobacco chewing was more common in the south (28%) compared to the north (2%). Smoking manufactured cigarettes was rare in both locations (3%) but smoking beedies was higher in the north (39% compared to 9% in the south). Chewing tobacco may have a more adverse effect on ascorbate levels compared to tobacco smoking because the tobacco quid is held in the mouth for a longer period of time but experimental data are not available.

Use of biomass fuels was associated with vitamin C deficiency. The smoke from combustion of biomass fuels includes small respirable particles, carbon monoxide, nitrogen formaldehyde and polyaromatic hydrocarbons. Since many of the constituents of biomass fuels are also found in tobacco smoke [20] it is likely that other adverse effects of biomass fuels on vitamin C are similar to those found for tobacco.

Seasonal differences in vitamin C deficiency varied between the north and south reflecting the different climatic and agricultural patterns across the sub-continent. In the north, the highest PRRs were observed for the main monsoon period (June to September) compared to the winter. Poor nutritional status in the monsoon months and higher dietary intakes of vegetables in the winter period have been reported from studies in the north of the sub-continent [21], [22]. In contrast the monsoon is lighter and later in the south of India. Dietary vitamin C levels also varied by season especially in the north (median of 30.6 mg/day in the winter compared to 15.7 in June to September). In the south the median values for these periods were 32.1 and 34.8 respectively.

Dietary vitamin C intakes from other studies in India [8], [23] are considerably lower than observed in western populations. However comparison of intakes of dietary vitamin C across studies and populations is limited by differences in the dietary assessment method and the availability of vitamin C data in Food Composition Tables for foods consumed in specific populations including values by cooking methods. We used a single 24 hour recall and the ICMR food composition tables which provide values of vitamin C from food items common to the Indian population. A limitation of these tables is that the values are based on raw foods. Loss of vitamin C occurs with heating and therefore dietary vitamin C in our study and in other studies in India is probably overestimated by at least 25% since the most common method of food preparation in the Indian population is cooking by heat [24], [25]. Although dietary vitamin C intakes are a major determinant of plasma vitamin C levels, this is difficult to demonstrate other than in tightly controlled experimental conditions [26]. In population surveys, dietary assessment methods including 24 hour recall show only moderate correlations with plasma vitamin C. A meta-analysis of studies in high income countries reported correlations between dietary vitamin C from diet recall (ranging from one day to 12 days) and plasma vitamin C of 0.46 [27]. The correlation was similar for one day to longer recall, in studies excluding supplement users, and was higher in women (r = 0.44) than men (r = 0.36). In our study the correlation coefficient between diet and plasma vitamin C was much lower (r = 0.20) and did not vary by sex. The authors of the meta-analysis concluded the moderate correlations observed might be influenced by factors including bioavailability, food processing and storage and recall errors by participants. These limitations are also applicable to our study.

Since Vitamin C is degraded by factors such as light, temperature (above 4°C) and oxidation, considerable care is required in the collection and processing of samples [28]. We collected blood in subdued lighting in vacutainer tubes containing the chelating agent EDTA to prevent the continued oxidation of vitamin C from metal ions and stored the tubes in a 4°C fridge for up to 2 hours before cold centrifugation and stabilization with MPA. Greater degradation of vitamin C with EDTA compared to heparin treated samples has been reported [29], [30]. In a study of 5 people with paired samples analyzed immediately after collection using the FRASC method, the mean ascorbate was around 50 µmol/L in the EDTA samples compared to around 80 µmol/L in the heparin samples [29]. Karslen et al found no significant difference between heparin and EDTA as anticoagulant when baseline ascorbate levels measured by HPLC were compared, with a mean 2.8% lower level of vitamin C in EDTA samples [30]. Delayed ascorbate measurement with samples left at room temperature showed greater degradation especially for EDTA samples (e.g. 10% loss at 2 hours compared to 5% heparin). Heparin samples stored at 4°C for 2 hours showed <1% degradation in MPA acidified plasma, compared to 5.6% degradation in non-acidified plasma and 10% loss for storage for 24 hours. The equivalent data for EDTA was not collected. In contrast, Ching et al in a study of 10 people reported that samples treated by heparin, centrifuged and acidified and measured by HPLC had 7% less ascorbate than EDTA samples treated the same way [31]; ascorbate loss was also significantly greater in heparin samples following a 2 hour delay in separation followed immediately by centrifugation and acidification (median 18% loss for heparin compared to 7% for EDTA). Although results from these small studies are not consistent with respect to heparin compared to EDTA and show considerable intra individual variation, all studies confirm the importance of refrigeration at 4°C for as short a period as possible, followed by immediate cold centrifugation and acidification. Once samples are frozen, plasma ascorbate is stable over long term storage at −70°C [32]. In our study the median storage time was just over one year. Although we had a clear protocol for the collection and processing of samples and laboratory staff were trained to follow the protocol, we cannot exclude that errors may have occurred leading to loss of vitamin C from the samples. Vitamin C showed typical patterns observed consistently in other studies, such as lower levels in men, in tobacco users, those with indices of poor nutrition, lower socio-economic status and an inverse association with age [15]. It is unlikely that these patterns would be preserved if the blood samples had degraded randomly but a systematic loss would lead to an over estimation of the prevalence of vitamin C deficiency. Quantifying the possible ascorbate loss in our study is uncertain but based on the literature reviewed above [29], [30], [31], [32] we might expect only minor degrees of ascorbate loss (possibly up to 10%) due to pre-analytical factors such as use of EDTA, and delays in centrifugation and freezing of samples in view of the sample handling protocol described in this study. However we acknowledge that the losses might be greater since we did not have any formal methods of quality assurance to ensure the protocol was followed. The levels for participants in north India in the present study were very similar to those in a small feasibility study we conducted previously in Haryana [33].

We had only a single measurement of plasma and dietary vitamin C and were unable to ascertain the effects of within person seasonal changes. Our response rates were acceptable (75%) and apart from age there was no response bias in sex or socio-economic status. Since vitamin C deficiency increased with age the prevalence of vitamin C deficiency might be underestimated.

Our population was primarily rural or from small towns, characterized by low BMI, high tobacco and biomass fuel use and low intakes of dietary vitamin C. In 15% the mid-upper arm circumference values were indicative of moderate to severe malnutrition. Our results may not apply to middle aged and younger people, city dwellers or high income groups and studies are required in these groups.

In addition to low dietary intakes of vitamin C, low plasma levels of vitamin C in India may also reflect haptoglobin (Hp) allele status (Hp1 or Hp2). The Hp2 -2 phenotype is substantially higher in India (around 70–80%) compared to populations of European ancestry (30–40%), and conversely Hp1-1 is much lower, less than 3% in India compared to around 15–20 % in Europeans [34]. Studies in Europeans have reported around 20% lower plasma vitamin C levels in those with the Hp2-2 polymorphism compared to those with Hp 1-1 [35]. A study of University of Toronto non-smoking students found vitamin C deficiency in 17% of those with Hp2-2 compared to 11% of those with either Hp 1-1or 2-1. The risk of deficiency in those with low dietary intakes of vitamin C (below recommended intakes) was modified by Hp status; from an OR of 1.7 for Hp1-1 or 2-1 to an OR of 4..8 for HP2-2 [36]. These data suggest that the effect of Hp2-2 may be greatest when dietary intakes of vitamin C are low. No data are presently available in India on vitamin C and haptoglobin polymorphism. An important function of haptoglobin is to bind haemoglobin preventing peroxidation by free iron; the observation of lower vitamin C levels in Hp2-2 individuals with lower haptoglobin may reflect the increased depletion of vitamin C due to reduction of free iron [37]. Haptoglobin polymorphisms might also explain in part the lower levels of vitamin C reported for South Asians in the UK compared to those of European or African ancestry [38] or for Indians in Singapore compared to Chinese [39]. However the differences in plasma vitamin C between ethnic groups in these studies were not large and the mean levels were much higher than in our study population. The studies on vitamin C in Indian ethnic groups in Singapore and the UK are in the settings of high income countries. Indians in these settings are characterized by BMIs in the normal to overweight range, more central obesity and higher dietary energy intakes. Although data are sparse it is likely that dietary intakes of vitamin C in Indians are also higher in high income settings, probably reflecting better nutrition of the Indian ethnic groups in contrast to our study participants. In a study in the UK, children of Indian ethnicity had slightly lower dietary intakes compared to white European children but both groups had intakes well above the recommended intakes for their age group [40]. Currently there are limited data on other genetic modifiers of vitamin C levels [41], [42] and no studies have been carried out in India.

The majority of previous reports on vitamin C deficiency from population based studies have taken place in the UK or North America. In these studies the prevalence of vitamin C deficiency ranged from 26% of men and 14% of women aged 25 to 74 years in the Glasgow MONICA study[6] ,25% of men and 16% of women aged 19 years and over in the UK Low Income Diet and Nutrition Survey[4], 14% of nonsmoking women and men aged 20–29 years in the University of Toronto campus [2]. In two waves of the US Nutrition and Health Examination Study of people aged 20 years and over, vitamin C deficiency was reported in 18% of men and 12% of women for 1998–1994 [3], reducing to 10% and 7% in the 2003–2004 survey; the prevalence for those in the lower income groups was double that of the high income groups at both time periods [5]. Only two studies have been conducted outside high income countries including one from India. A nationally representative population study from Mexico reported a 40% prevalence of deficiency in women of childbearing age [43]. Men and older people were not included in the study. In a small study of 322 people aged 20–50 years from western India, vitamin C deficiency was found in 9.6% of men and 13.0 % of women, and just over a half had levels in the sub-optimal range [44].

In conclusion, we found vitamin C deficiency in a substantial proportion of the older population in two settings in north and south India. Only 10% of those in the north and a quarter of those in the south met the criteria for adequate levels. Our results are relevant to current debates about the control of non-communicable diseases in India. Low fruit and vegetable intake, tobacco use and biomass fuels contribute respectively the third, fourth and fifth ranked risk factors associated with mortality and disease burden in India [45]. Our results show that low dietary vitamin C intakes (reflecting low fruit and vegetable intake), tobacco use and biomass fuels are risk factors for vitamin C deficiency and add to the evidence on the health consequences of these risk factors. In poor communities, such as described in our study, consideration needs to be given to measures to improve the consumption of vitamin C rich foods and to discourage the use of tobacco. This includes a raft of measures including agricultural and tobacco policy, promoting awareness in communities through education and employment of local dieticians. The growing proportion of older people in India also highlights the importance of better information on the nutritional status of this age group.
